# Career anxiety in the age of artificial intelligence: Survey data of university students in Bangladesh

**DOI:** 10.1016/j.dib.2026.112924

**Published:** 2026-06-05

**Authors:** Shahrin Islam, Bibhas Roy Chowdhury Piyas, Fatama Jannat Tisha, Humayun Kabir Nayem, Sadia Rahman, Bijoy Roy Chowdhury Preenon, Shazzad Hossen

**Affiliations:** a Department of Software Engineering, Daffodil International University, Dhaka 1216, Bangladesh; b Department of Computer Science and Engineering, Chittagong University of Engineering and Technology, Chattogram 4349, Bangladesh; c Department of Electrical and Electronic Engineering, Independent University, Dhaka 1229, Bangladesh; d Department of Computer Science and Engineering, Daffodil International University, Dhaka 1216, Bangladesh

**Keywords:** Career anxiety, Artificial intelligence, AI adoption, Labor market perception, Job insecurity, Employment uncertainty, Employability skills, AI-driven job insecurity

## Abstract

Career anxiety has become a significant issue in today’s world, particularly in developing countries where job opportunities are scarce compared to the rising number of university graduates. The rapid advancement of artificial intelligence (AI) has heightened this anxiety by reshaping labour markets and automating an increasing number of job roles. Although numerous studies have examined general career anxiety among students, research particularly addressing career anxiety triggered by AI remains scarce. To address this gap, this article presents findings from an online survey conducted across five prominent public and private universities in Bangladesh. The survey examined students’ perceptions of artificial intelligence (AI) and its potential impact on their future careers. A total of 3156 students from various departments and academic years participated in the survey. The study covers students’ knowledge of AI, frequency of AI tool usage and perceptions of AI as a potential threat to their chosen professions and future employment. The dataset also records self-reported levels of career anxiety and strategies students use to prepare for an AI-driven job market. Statistical analyses and visualizations reveal variations of career anxiety across age, gender, academic year, department and AI knowledge level, as well as document the specific AI tools students perceive as potential threats to future employment. The dataset provides meaningful guidance for educators and policymakers in reshaping curriculum to prepare students for new workforce realities. It may also act as a foundation for future research in predictive modelling, labour market analysis, behavioural patterns and human–AI collaboration.

Specifications Table

**Table:** 

Subject	Computer Science
Specific subject area	Career Anxiety due to AI; Artificial Intelligence; AI Adoption; Human–AI Collaboration; Labor Market Perception; Educational Research
Type of data	Survey data (tabular, CSV format)
Data collection	Data were curated from university students across different semesters that resulting in a total of 3156 responses for this study. The survey tool was carefully designed, validated and implemented using Google Forms. We invited Participants to respond via email. To provide context, a seminar was organized to introduce students to AI, covering its applications, tools, potential threats, opportunities and its implications for career development. After the seminar, students from multiple departments were encouraged to complete the survey. Additionally, the questionnaire was distributed across classrooms, the library, and the student lounge to maximize accessibility and participation.
Data source location	The data were collected from students of several departments of Daffodil International University, Jahangirnagar University, American International University-Bangladesh, University of Dhaka and Chittagong University of Engineering and Technology. All of these universities are located in Bangladesh.
Data accessibility	Repository name: Mendeley Data Data identification number: https://doi.org/10.17632/r3xh8sd764.3 Direct URL to data: https://data.mendeley.com/datasets/r3xh8sd764/3 Citable Zenodo link for the GitHub repository: https://doi.org/10.5281/zenodo.18461007
Related research article	None

## 1 Value of the Data

• This is the first publicly available large-scale survey dataset from Bangladesh that particularly investigates university students’ career anxiety in the context of rapid advancements in artificial intelligence (AI) which addresses a notable gap in existing research.
• Although the dataset consists of a limited number of core variables, these are deliberately structured and conceptually interconnected to assess career anxiety within a specific context. The variables capture AI knowledge, usage patterns, perceived occupational threat and career anxiety levels which makes the dataset valuable for other researchers by providing an interpretable framework to examine AI-induced career anxiety.
• The large and diverse sample size (N = 3156) covering multiple universities, disciplines and academic levels, enhances the statistical reliability of the dataset and supports subgroup and comparative analyses.
• The dataset can serve as a valuable resource for interdisciplinary research in psychology, education, computer science and labour market studies. It is particularly useful for predictive modelling, behavioural analysis and examining human–AI interaction.
• It offers practical insights for educators, policymakers and institutions to better understand AI-related career concerns among students and to support curriculum development, career guidance and skill-building initiatives aligned with future workforce demands.
• This dataset can be used for comparison with future surveys to track how student perceptions of AI and career anxiety evolve over time as AI technologies advance and job markets shift.

## 2 Background

Artificial intelligence is reshaping industries around the world, altering job roles and changing the skills needed for future employment. While AI-driven automation contributes to higher productivity and new forms of innovation, it has also raised concerns among young people preparing to enter the workforce. According to a number of studies, young professionals and students are becoming increasingly concerned about how developing technology could disrupt career paths and diminish the value of their credentials. Skalka et al. [1] surveyed 1146 university students across countries to evaluate AI literacy, readiness, and anxiety, tracking global trends in AI perceptions. Smit et al. [2] explored master’s students’ perspectives on AI and generative AI in South African higher education. In the context of human-AI interaction research, Shank et al. [3] examined self-reported encounters with AI systems exhibiting human-like traits, revealing how people attribute mind, emotions, and intentions to AI. Focusing on AI integration in higher education, Luthfia et al. [4] analyzed data from 535 Indonesian students, highlighting AI usage, attitudes, and motivations shaping AI adoption. Chen et al. [5] revealed that AI anxiety can enhance motivated learning among undergraduates with AI self-efficacy. Drawing on data from 455 Chinese students, the authors of [6] demonstrated that positive AI attitudes and literacy alleviate job-seeking anxiety, whereas [7] presented a contrasting perspective, indicating that AI-related anxiety undermines work passion through emotional exhaustion. Study [8] showed that individual innovativeness mediates the link between career engagement and AI anxiety, while [9] found that demographic and contextual factors influence AI-related anxiety and fear by surveying over 200 science students and professionals. Beyond educational and psychological perspectives, advancements in natural language processing and sentiment analysis have enabled researchers to better understand human emotions and cognitive behaviour. [10,11] demonstrate how sentiment analysis and text classification techniques can effectively capture nuanced emotional expressions. Ge et al. [12] examined 716 Chinese college students and found that AI awareness has a dual effect, simultaneously increasing employment anxiety and promoting career exploration. Expanding this line of research, this dataset contributes to understanding AI-induced career anxiety among Bangladeshi university students and offers insights into their perceptions, preparedness, and adaptation strategies for an AI-driven job market.

## 3 Data Description

The dataset, “Career Anxiety due to Artificial Intelligence: Survey Data of University Students in Bangladesh,” comprises responses from 3156 students representing multiple academic departments and semesters across several well-established public and private universities in Bangladesh. The survey was carried out using Google Forms. Before participation, students attended an introductory seminar covering artificial intelligence, its tools, potential risks and benefits and its relevance to career planning for informed participation and engagement. Moreover, the questionnaire was distributed in different classrooms, the library and the student lounge to maximize accessibility and participation. Table 1 provides a general overview of the dataset covering column descriptions, data types, and example values for each variable.

**Table 1:** Overview of the column in the dataset.

Column Name	Description	Data Type	Example Values
Participant_id	Participant Response ID	Text	Farjan Ahmmed
University	Name of the university	Text	Chittagong University of Engineering and Technology
Department	Academic department of the student	Text	Department of Software Engineering
Age	Age of the student in years	Integer	22
Gender	Gender of the participant	Text	Male
Academic_year	Academic year of study	Text	3rd Year
Ai_knowledge	Self-reported knowledge of AI (e.g., Low, Medium, High)	Text	High
Ai_tools_used	Three most frequently used AI tools	Text	ChatGPT, Deepseek, Claude
Ai_future_perspective	Students’ Perception of the Future of AI	Text	AI will significantly replace human jobs and create major challenges
Career_path	Career field the student wishes to pursue	Text	Machine Learning Engineer
Ai_tool_perception	Which AI tools are perceived as a threat to the chosen career	Text	ChatGPT
Ai_replace_jobs	Opinion on whether AI can replace human jobs one day	Text	Partially agree
Ai_takeover_time	Estimated timeline when AI could completely replace human jobs	Text	6–10 years
Career_anxiety	Self-reported level of career anxiety due to AI	Text	Moderate

**Fig. 1:**
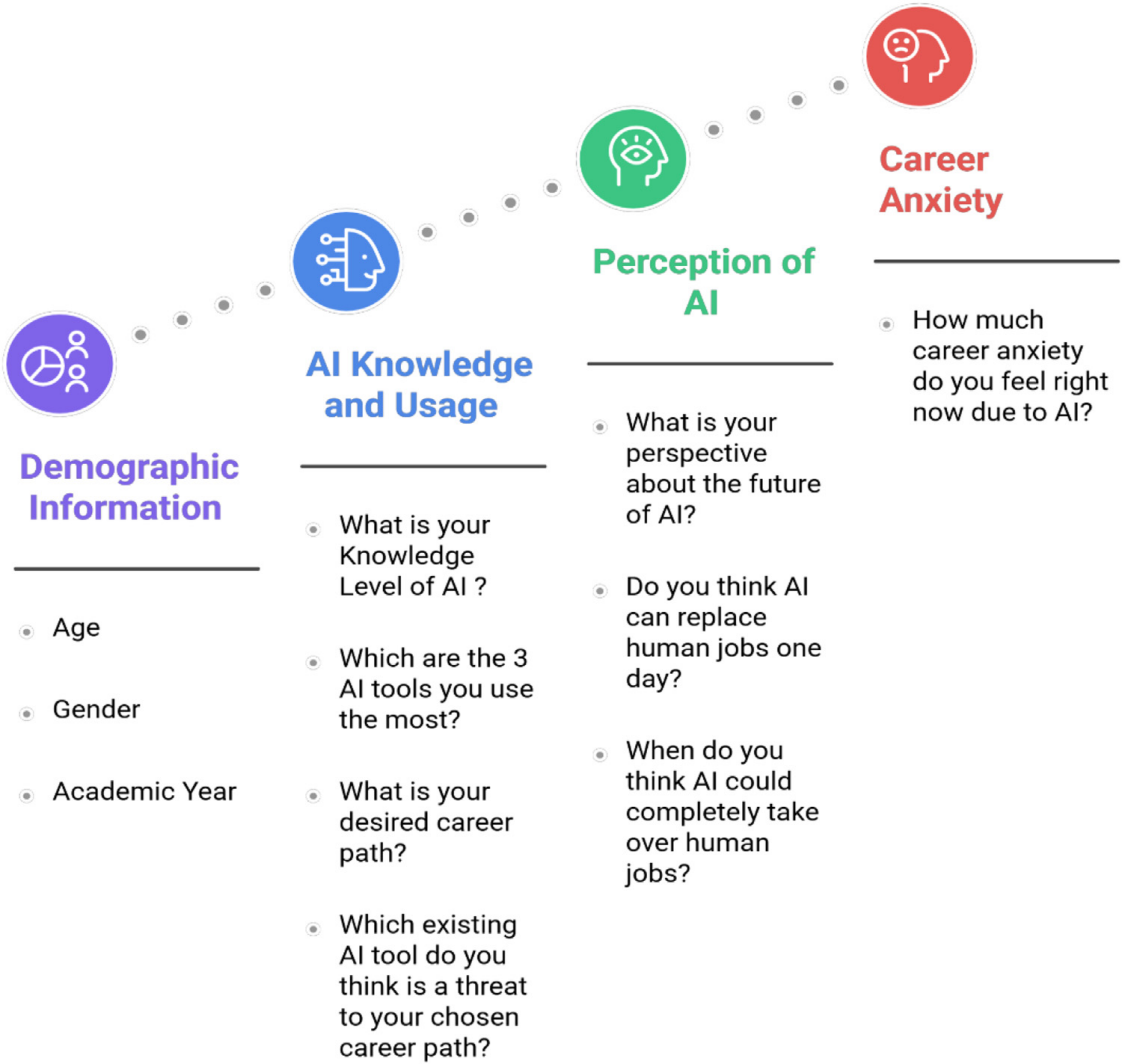
Thematic organization of the developed dataset.

Fig. 1 demonstrates the thematic organization of the developed dataset which includes demographic information, knowledge and usage of AI, perspectives on AI and its threats as well as career anxiety related to AI. This section provides a detailed explanation of each component.

### 3.1 Demographic Data

**Fig. 2:**
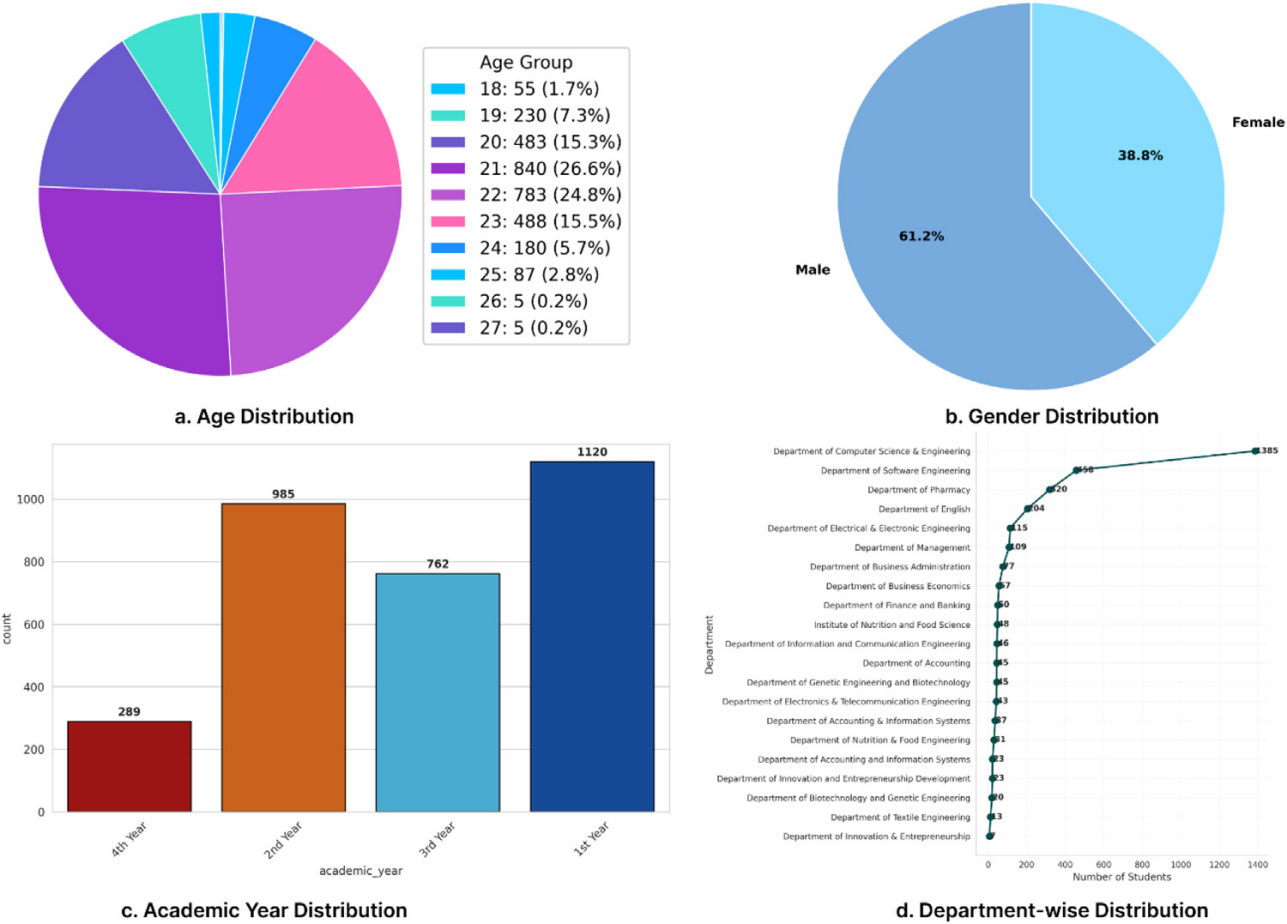
Demographic distribution of participants.

The dataset begins with the demographic information of the participants, including their department, age, gender, and current academic year. Fig. 2 depicts that the majority of participants fall within the age range of 20–23 years, with a notably high participation from the students of 1st and 2nd academic years. Male students participated in greater numbers compared to female students. Additionally, most respondents were from the Departments of Computer Science and Engineering (CSE) and Software Engineering (SWE), which can be associated to the higher relevance of the research theme to these departments and the larger student population in these departments compared to others.

### 3.2 Knowledge and usage of AI

To keep up with the fast-changing job market shaped by new technologies, it is important for students to have a solid understanding of AI. It also helps students reduce their career-related anxiety. We collected self-evaluated AI knowledge levels from students on a scale ranging from high to none. The results shown in Table 2 indicate that around 75.22% of the students consider their AI knowledge to be at a medium level and believe they need to attain additional skills to get prepared for the future market.

**Table 2:** AI knowledge level.

Knowledge Level	Count	Percentage (%)
High	214	6.78
Medium	2374	75.22
Low	523	16.57
None	45	1.43

**Fig. 3:**
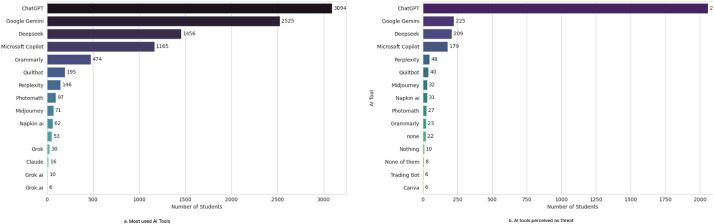
Usage and Perception of AI Tools.

Fig. 3 extracts a paradoxical relationship where the AI tools that students use frequently are also perceived as potential career threats. The study indicates that students commonly use tools such as ChatGPT, DeepSeek, and Gemini to solve their everyday tasks but they simultaneously perceive these tools as threats to their future careers.

### 3.3 Perspectives on AI and career threats

Table 3 presents participants’ perceptions of AI based on five levels of detailed survey questions. The questions examine students’ perspectives on the future of AI, covering both its opportunities and potential risks. The survey indicates that around 43% of students believe AI will replace certain human jobs while also creating new opportunities. However, the pole of the negative responses are comparatively heavier, showing that most students still do not view AI as a career opportunity but rather perceive it as a potential career threat.

**Table 3:** Perspective on the future of AI.

Statement	Count	Percentage (%)
AI will completely take over human jobs and pose serious threat	487	15.43
AI will significantly replace human jobs and create major challenges	507	16.06
AI will replace some human jobs but also create new opportunities	1358	43.03
AI will mostly assist humans, with limited job replacement	375	11.88
AI cannot take over human activities and will be helpful	429	13.59

### 3.4 Career anxiety due to AI

Participants rated their current level of career anxiety due to AI on a four-point scale ranging from “no anxiety” to “high anxiety” in Table 4. The results show that 51.77% of students stated a moderate level of anxiety, while 14.61% rated their anxiety as high. With the ever-changing nature of AI, this level of anxiety may continue to increase in the future.

**Table 4:** Career anxiety levels.

Anxiety Level	Count	Percentage (%)
High	461	14.61
Medium	1634	51.77
Low	648	20.53
No Anxiety	413	13.09

## 4 Experimental Design, Materials and Methods

This study used a quantitative correlational survey to examine how university students perceive the increasing influence of artificial intelligence and to what extent it affects their career anxiety. Additionally, it determines how students in various fields perceive the potential effects of AI on their future careers.

Participants were undergraduate students from five prominent universities of Bangladesh. Students from diverse departments and academic years were encouraged to participate in order to gather a wide range of perspectives. In total, 3156 responses were collected from students enrolled in various semesters. Participation was voluntary and only the submissions of students who gave informed consent were included in the final analysis.

A standardized Google Forms questionnaire that was intended to record both quantitative and qualitative replies was used to gather data. Before starting data collection, an awareness seminar was organized at the university auditorium to introduce students to the concept of AI, its applications, potential risks and career implications. After the session, the survey link was distributed via institutional email and shared at various student gathering points, including classrooms, library and student lounge to maximize participation. Participants were informed about the purpose of the research. The survey took approximately 5–7 min to complete for each participant.

### 4.1 Survey instrument

To assess the career anxiety of university students, a structured survey instrument was developed consisting of four segments: demographic information, AI knowledge and usage, perception of AI and career anxiety. For each segment, specific items were included in the form of survey questions, along with defined response options and corresponding encoding strategies used in the final dataset. This section provides a detailed overview of each measure, including the items used and their development, indicating whether they were designed for this study or adapted from existing literature.

#### 4.1.1 Demographic information

Demographic data were collected to describe the background characteristics of the participants, including age, gender, academic year. Table 5 presents an overview of the demographic survey items.

**Table 5:** Overview of survey items on demographics.

Items	Response Options	Encoding Strategies	Source
Age	Open-ended	Not encoded	Adapted from Skalka et al. [1], Smit et al. [2]**,** Luthfia et al. [4], Lund et al. [9]
Gender	Open-ended	Not encoded	Adapted from Skalka et al. [1], Smit et al. [2]**,** Luthfia et al. [4], Lund et al. [9]
Academic Year	Open-ended	Not encoded	Adapted from Skalka et al. [1], Smit et al. [2]**,** Luthfia et al. [4], Lund et al. [9]

#### 4.1.2 AI knowledge and usage

Survey items were developed for this study to assess participants’ level of AI knowledge and to capture their usage of AI tools in everyday activities. Table 6 provides an overview of survey items on AI knowledge and usage

**Table 6:** Overview of survey items on AI knowledge and usage.

Items	Response Options	Encoding Strategies	Source
What is your Knowledge Level of AI?	High	Coded as 3	Developed by authors for this study
	Medium	Coded as 2	
	Low	Coded as 1	
	None	Coded as 0	
Which are the 3 AI tools you use the most?	ChatGPT	Not Encoded	Adapted from Smit et al. [2], Luthfia et al. [4]
	Google Gemini		
	Microsoft Copilot		
	Midjourney		
	Deepseek		
	Napkin AI		
	Perplexity		
	Photomath		
	Quiltbot		
	Grammarly		
	Other		
What is your desired career path?	Open-ended	Not Encoded	Developed by authors for this study
Which existing AI tool do you think is a threat to your chosen career path?	ChatGPT	Not Encoded	Developed by authors for this study
	Google Gemini		
	Microsoft Copilot		
	Midjourney		
	Deepseek		
	Napkin AI		
	Perplexity		
	Photomath		
	Quiltbot		
	Grammarly		
	Other		

#### 4.1.3 Perception of AI

Survey items were developed to assess participants’ perceptions of the future of AI, including their beliefs about AI’s potential to replace human jobs and their expectations regarding the timeline of such developments. Table 7 shows an overview of the survey items on perceptions of AI.

**Table 7:** Overview of survey items on perception of AI.

Items	Response Options	Encoding Strategies	Source
What is your perspective about the future of AI?	I strongly believe AI will completely take over human jobs and pose a serious threat to humanity.	Coded as 4	Adapted from Luthfia et al. [4]
	I believe AI will significantly replace human jobs and create major challenges.	Coded as 3	
	I think AI will replace some human jobs but also create new opportunities.	Coded as 2	
	I believe AI will mostly assist humans, with limited job replacement.	Coded as 1	
	I strongly believe AI cannot take over human activities and will be a helpful tool for humans.	Coded as 0	
Do you think AI can replace human jobs one day?	Fully	Coded as 2	Adapted from Chen et al. [7]
	Partially	Coded as 1	
	No	Coded as 0	
When do you think AI could completely take over human jobs?	1–5 years	Coded as 5	Developed by authors for this study
	6–10 years	Coded as 4	
	11–20 years	Coded as 3	
	21–50 years	Coded as 2	
	50+ years	Coded as 1	
	Never	Coded as 0	

#### 4.1.4 Career anxiety

A survey item was used to capture the self-reported level of career anxiety experienced by participants due to the rapid advancement of artificial intelligence. Table 8 presents an overview of survey items on career anxiety.

**Table 8:** Overview of survey items on career anxiety.

Items	Response Options	Encoding Strategies	Source
How much career anxiety do you feel right now due to AI?	High	Coded as 3	Adapted from Li et al. [6], Lund et al. [9]
	Medium	Coded as 2	
	Low	Coded as 1	
	No Anxiety	Coded as 0	

### 4.2 Demographics and career anxiety

**Fig. 4:**
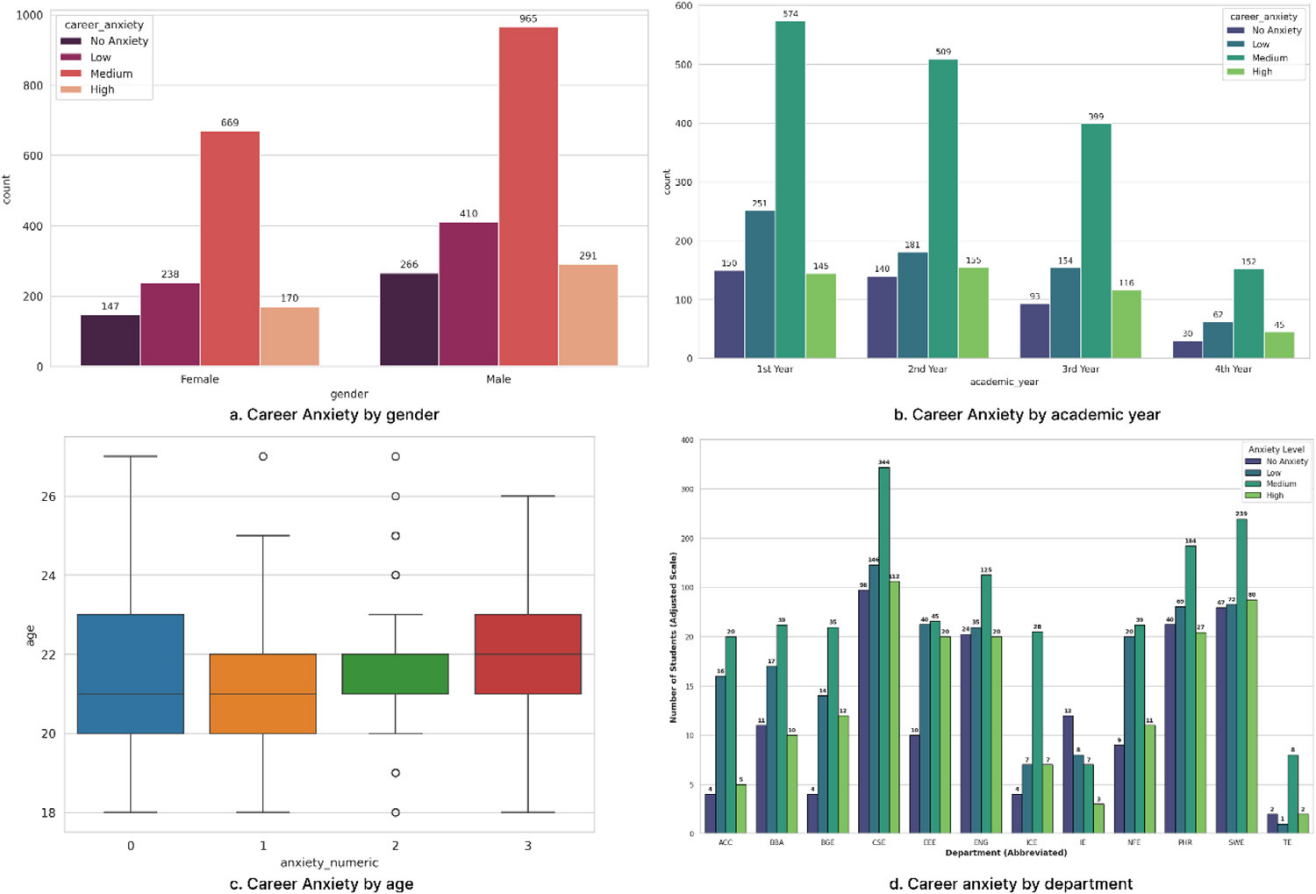
Relationship between demographic factors and career anxiety.

Fig. 4 shows how career anxiety is distributed across different demographic variables, including gender, academic year, age and department. The data indicate that moderate levels of career anxiety are reported across all academic years. Career anxiety levels vary across departments, with higher counts observed in the Computer Science and Engineering and Software Engineering departments. In terms of gender, male students report higher counts of career anxiety compared to female students. Additionally, higher levels of career anxiety are observed among students aged 22 and above.

### 4.3 AI knowledge level and career anxiety

**Fig. 5:**
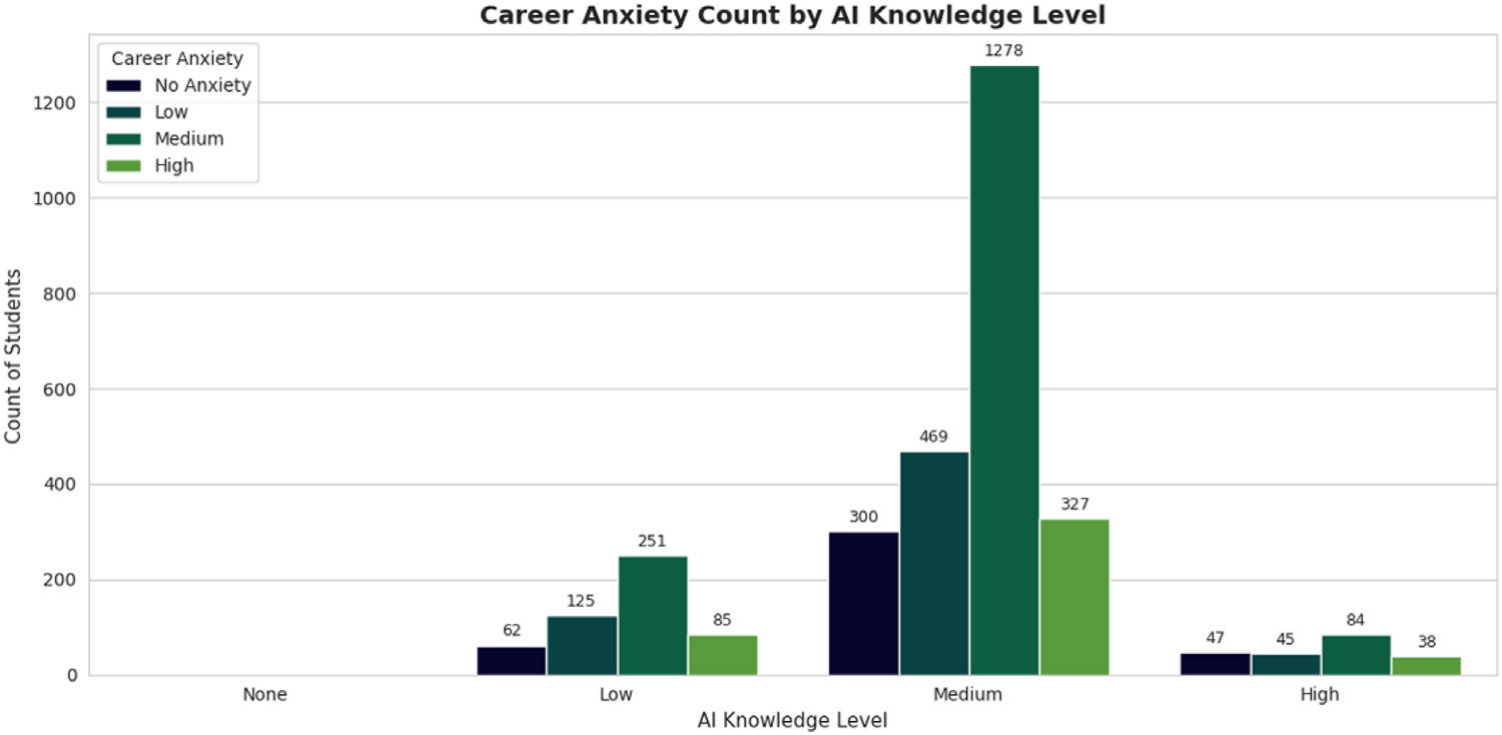
Relationship between demographic factors and career anxiety.

Fig. 5 presents the distribution of career anxiety levels across different levels of AI knowledge among students. Students with medium AI knowledge show the highest counts across the career anxiety categories. In comparison, students with low and high AI knowledge show relatively lower counts of high anxiety. Students with no AI knowledge are represented in relatively small numbers across the categories. Overall, the figure illustrates variations in career anxiety across different levels of AI knowledge.

### 4.4 Career-specific AI anxiety

**Fig. 6:**
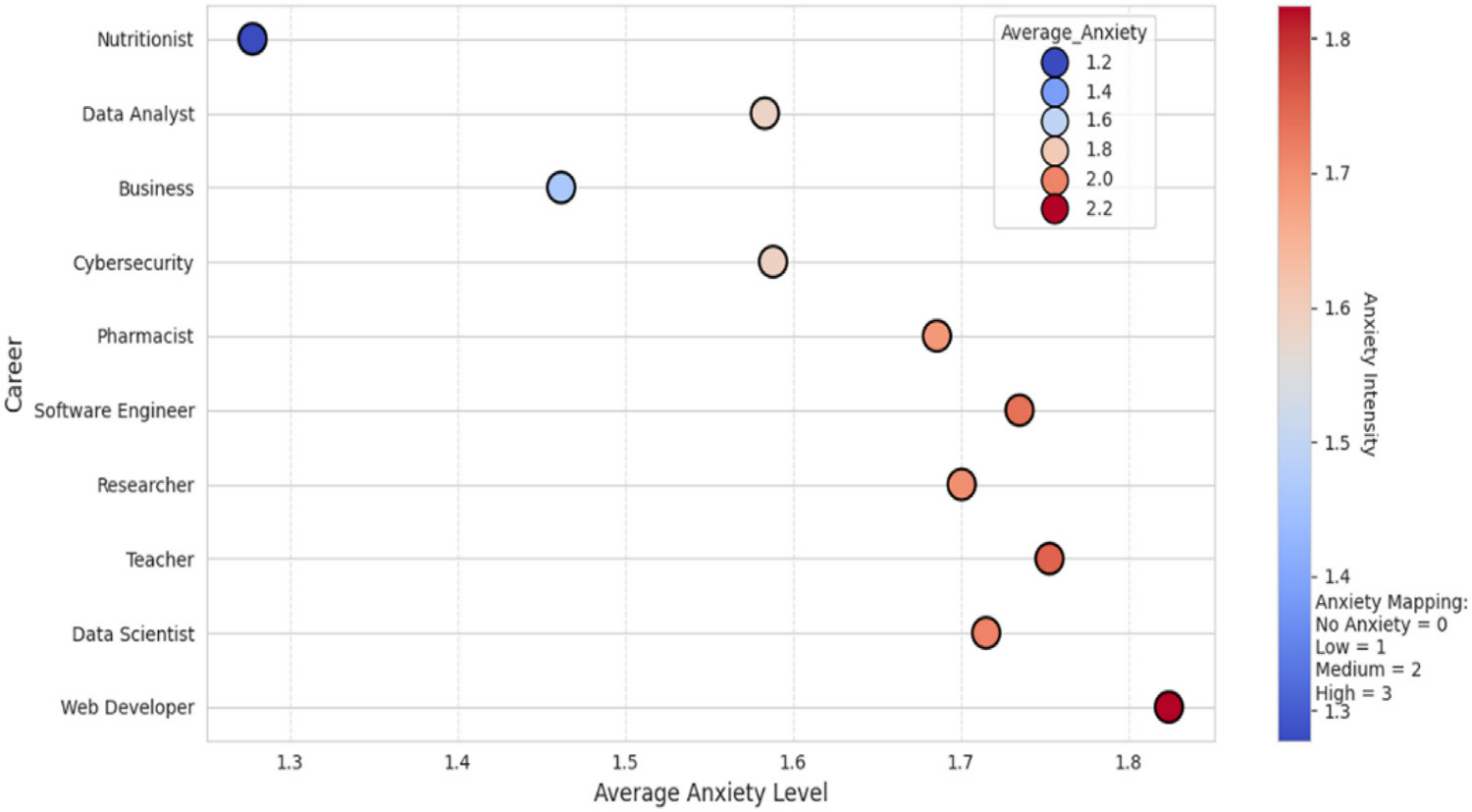
Career-wise distribution of anxiety levels.

Fig. 6 shows the average career anxiety levels across the ten most commonly selected career paths among students. The figure indicates that students aspiring to become web developers report the highest average anxiety levels. Higher average anxiety is also observed among students aiming for careers such as data scientist, researcher and software engineer. In contrast, lower average anxiety levels are observed among students pursuing careers such as nutritionist, business and data analyst. Overall, the figure presents variations in average career anxiety across different career choices.

### 4.5 Career anxiety and perceptions of AI job takeover

**Fig. 7:**
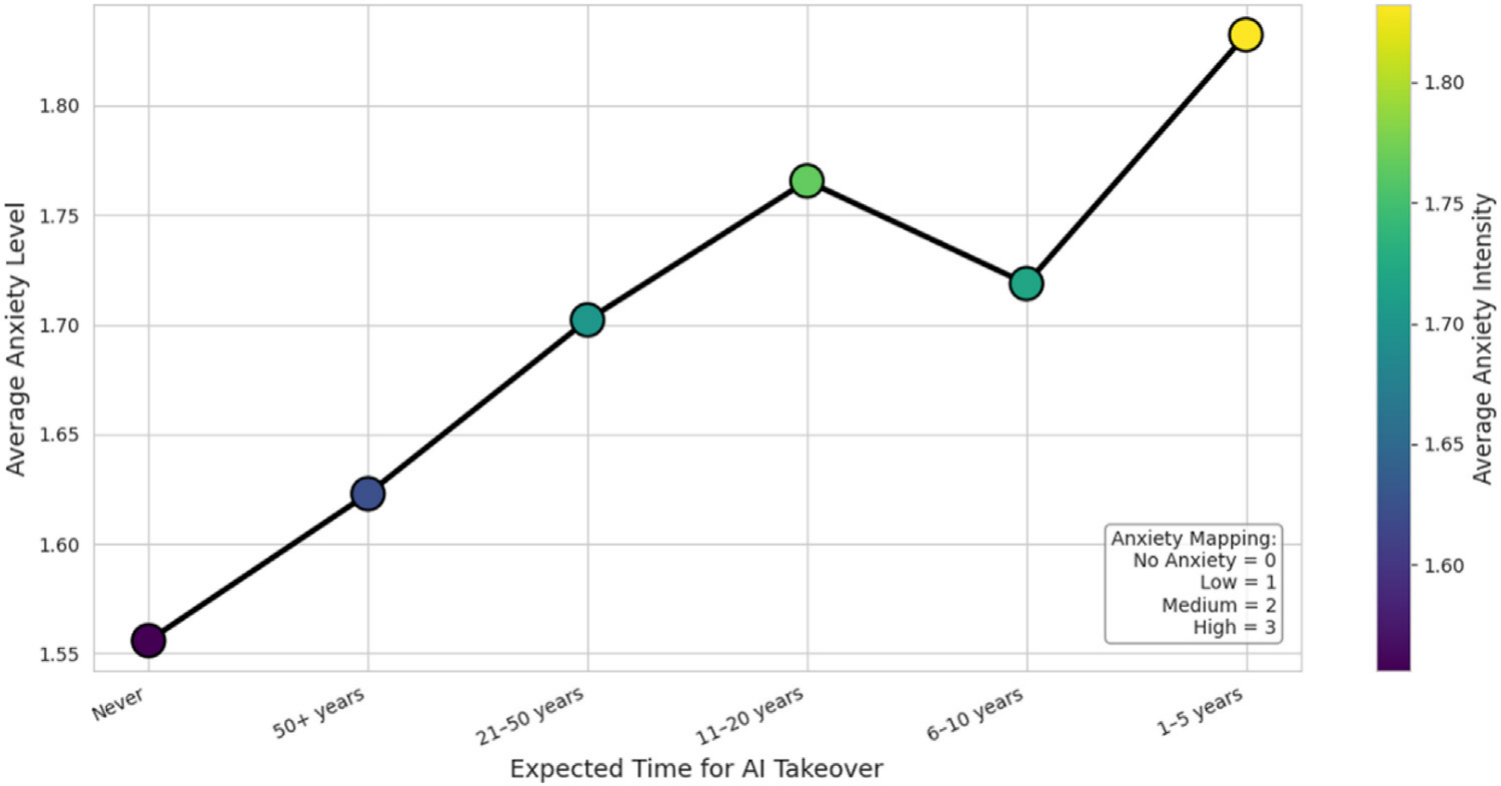
Career anxiety by perceived timeline of AI Job takeover.

Fig. 7 shows the variation in average career anxiety levels based on students’ perceived timeline for AI job takeover. Students who expect AI to replace human jobs within 1–5 years report the highest average anxiety levels. Lower average anxiety levels are observed among students who perceive longer timelines for AI takeover. Students who believe that AI will never replace human jobs show the lowest average anxiety levels. Overall, the figure illustrates differences in career anxiety across varying expectations of AI job replacement timelines.

## Limitations

While this study makes an effort to analyse career anxiety among university students due to rapid AI advancement, it has few limitations. Data were collected from a single country, which may not fully reflect the diversity of student perspectives elsewhere. Future research could broaden the dataset by including students from multiple universities across different countries to make it more balanced representation.

## Ethics statement

The dataset was collected following standard ethical research practices. Participants were clearly informed about the purpose and theme of the research. The survey included a consent section where participants could choose whether their responses could be used for research. Only responses from participants who gave consent were included in the dataset. All identifiable participant information was anonymized to ensure privacy and confidentiality. Verbal ethical approval was obtained from the appropriate institutional review boards to ensure compliance with ethical requirements. As the dataset is fully anonymized, non-sensitive and does not involve medical or vulnerable population data, formal ethics approval numbers are typically not issued for such studies. This practice is commonly followed for surveys involving highly sensitive or clinical data. These steps ensure responsible data use and compliance with legal and ethical research standards.

## Credit Author Statement

**Shahrin Islam:** Conceptualization, Data curation, Methodology, Software, Writing – original draft, Visualization. **Bibhas Roy Chowdhury Piyas:** Conceptualization, Data curation, Methodology, Software, Writing – original draft, Visualization. **Fatama Jannat Tisha:** Data curation, Methodology, Investigation, Validation, Writing – review & editing. **Humayun Kabir Nayem:** Data curation, Methodology, Investigation, Validation, Writing – review & editing. **Sadia Rahman:** Methodology, Investigation, Validation, Writing – review & editing. **Bijoy Roy Chowdhury Preenon:** Methodology, Investigation, Validation, Writing – review & editing. **Shazzad Hossen:** Methodology, Investigation, Validation, Writing – review & editing

## Data Availability

Mendeley DataCareer Anxiety in the Age of Artificial Intelligence: Survey Data of University Students in Bangladesh (Original data)

## Appendix Supplementary materials

Image, application 1
