# Supplementary material for: Career anxiety in the age of artificial intelligence: Survey data of university students in Bangladesh
Source: Data Brief. 2026 Jun 5;67:112924. doi: 10.1016/j.dib.2026.112924 (PMC13264088; doi:10.1016/j.dib.2026.112924)
Supplement: Supplementary file 1 [file mmc1.docx]

Dear Sir,

Please find below the required documents as suggested for submission:

- Citable Zenodo link for the GitHub repository containing complete questionnaire and source code - <https://doi.org/10.5281/zenodo.18461007>
